# How should studies using AI be reported? lessons from a systematic review in cardiac MRI

**DOI:** 10.3389/fradi.2023.1112841

**Published:** 2023-01-30

**Authors:** Ahmed Maiter, Mahan Salehi, Andrew J. Swift, Samer Alabed

**Affiliations:** ^1^ Department of Infection, Immunity & Cardiovascular Disease, University of Sheffield, Sheffield, United Kingdom; ^2^ Department of Radiology, Sheffield Teaching Hospitals, Sheffield, United Kingdom

**Keywords:** artificial intelligence, machine learning, cardiac MRI, segmentation, systematic review, quality of reporting

## Abstract

Recent years have seen a dramatic increase in studies presenting artificial intelligence (AI) tools for cardiac imaging. Amongst these are AI tools that undertake segmentation of structures on cardiac MRI (CMR), an essential step in obtaining clinically relevant functional information. The quality of reporting of these studies carries significant implications for advancement of the field and the translation of AI tools to clinical practice. We recently undertook a systematic review to evaluate the quality of reporting of studies presenting automated approaches to segmentation in cardiac MRI (Alabed et al. 2022 Quality of reporting in AI cardiac MRI segmentation studies—a systematic review and recommendations for future studies. *Frontiers in Cardiovascular Medicine* 9:956811). 209 studies were assessed for compliance with the Checklist for AI in Medical Imaging (CLAIM), a framework for reporting. We found variable—and sometimes poor—quality of reporting and identified significant and frequently missing information in publications. Compliance with CLAIM was high for descriptions of models (100%, IQR 80%–100%), but lower than expected for descriptions of study design (71%, IQR 63–86%), datasets used in training and testing (63%, IQR 50%–67%) and model performance (60%, IQR 50%–70%). Here, we present a summary of our key findings, aimed at general readers who may not be experts in AI, and use them as a framework to discuss the factors determining quality of reporting, making recommendations for improving the reporting of research in this field. We aim to assist researchers in presenting their work and readers in their appraisal of evidence. Finally, we emphasise the need for close scrutiny of studies presenting AI tools, even in the face of the excitement surrounding AI in cardiac imaging.

## Introduction

The development and application of artificial intelligence (AI) is an exciting frontier in radiology (1–3). AI tools promise automation of complex and time-intensive tasks, making them appealing in an era in which the demand and complexity of medical imaging are increasing. This is reflected in the recent rapid expansion in the number of studies presenting AI tools for imaging. However, there are several challenges that need to be overcome before AI can be implemented effectively in routine clinical practice (4). Transparency of model design, training and testing is critical for understanding the generalisability of tool but can be problematic where technologies are proprietary. Evaluating the performance of AI tools in relevant populations and environments is also an important step for determining their external validity. There is also growing awareness of ethical issues within the field. These include concerns about the risk of AI tools propagating human biases, including racial, that could cause discrimination for minority population groups (5–7). These challenges are inherently linked to the manner and quality in which studies of AI tools are presented.

The ability to compare evidence underpins modern medicine and necessitates that research is presented in a transparent, consistent and reproducible manner. Poor quality of reporting can contribute to research waste, hinder advancement of the field and limit clinical applicability. It is important for all stakeholders—including researchers, radiologists using AI tools, clinicians using AI-derived information and the public—to understand what constitutes high quality reporting. Structured tools have been proposed to assist the reporting of studies using AI, including the Checklist for Artificial Intelligence in Medical Imaging (CLAIM) (8).

## AI for segmentation in CMR

The demand for cardiac imaging is growing, and with it the appetite for automation. Cardiac MRI (CMR) allows non-invasive assessment of both cardiac anatomy and function. CMR can yield quantitative metrics (such as ventricular volumes, myocardial thickness and infarct sizes) that are of diagnostic and prognostic value. However, these measurements require the accurate delineation of anatomical structures on imaging, or segmentation. Those reading CMR studies have traditionally performed manual segmentation in order to derive these metrics—a process that is laborious, time-intensive and prone to interobserver variability. The ability to automate this process using AI methods has been the focus of an increasing number of studies in recent years (9–12).

In the broadest terms, AI automates processes traditionally performed by humans. Machine learning is a major branch of AI in which a program automatically identifies relevant features in data and adapts to improve its performance at a task. Machine learning encompasses a broad range of techniques, including deep learning and neural networks. In the context of segmentation in medical imaging, this involves a program learning to identify anatomical features in an image (such as the endocardium) in order to delineate structures (such as the cardiac chambers). Although the specific approaches and model designs are myriad, they have to date shared some similarities in their development. This typically involves three stages: training, validation and testing. During training, data is passed through the algorithm and the algorithm identifies features that enable it to undertake a task. In the validation stage, the algorithm is exposed to the unseen validation set and its performance at the task is determined. The algorithm is then adapted to optimise its performance and the training and validation steps are repeated until satisfactory performance is achieved and a final model is established. The model is then tested on new, unseen, data to yield its final performance results. This is a gross simplification of varied and complex processes, but is nonetheless important for contextualising how studies using AI are reported.

## The systematic review

**Figure 1:**
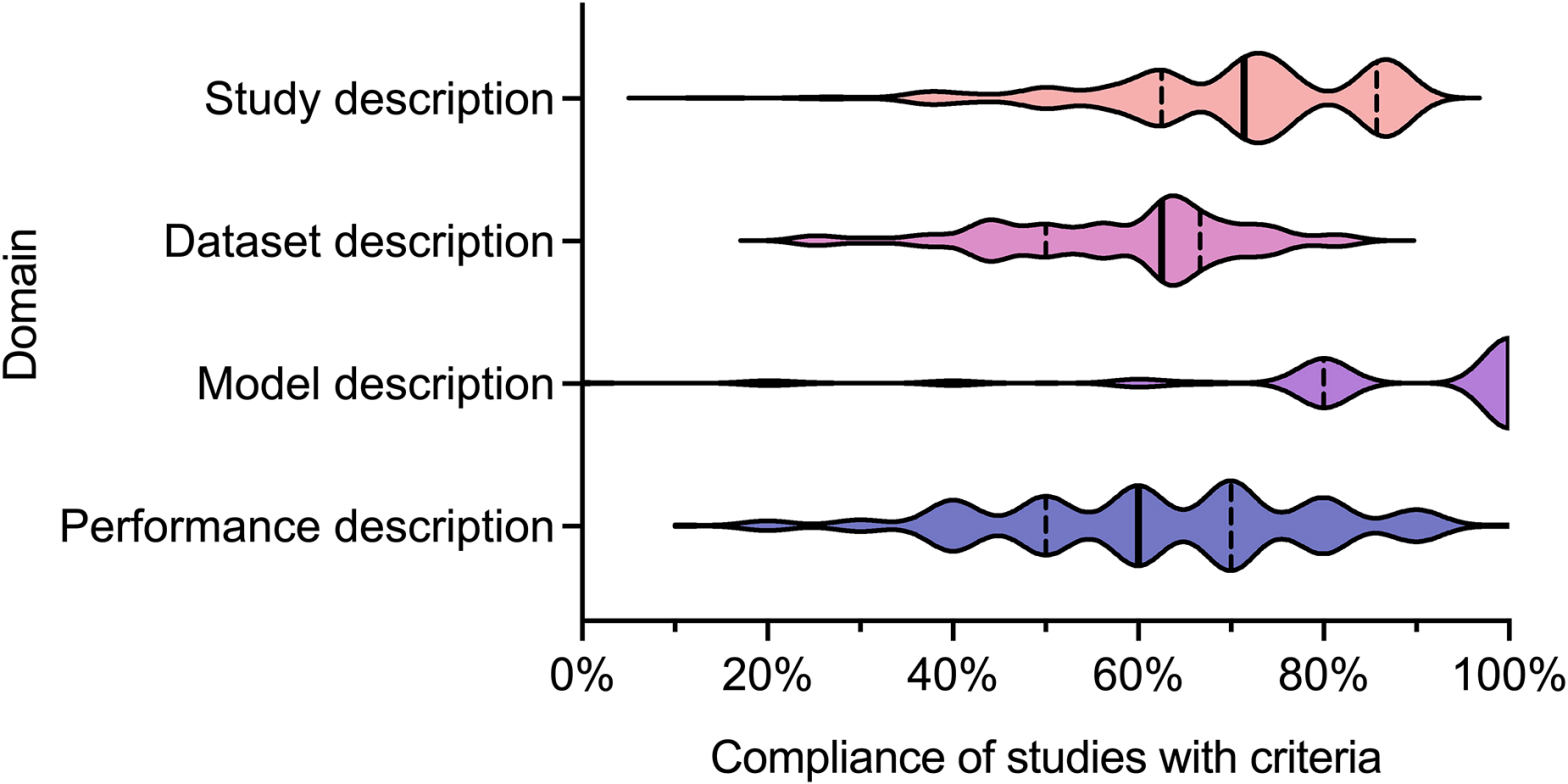
Violin plot indicating compliance of the 209 included studies with the CLAIM criteria, grouped into domains of study, dataset, model and performance description. Median (*solid line*) and 1st and 3rd quartile (*dashed lines*) values are indicated. From Alabed et al 2022 (13).

We recently undertook a systematic review of the quality of reporting of studies using AI methods for segmentation of structures on CMR (13). Studies presenting fully automated AI methods for the segmentation of cardiac chambers, myocardium or scar tissue on adult CMR images were eligible for inclusion. Included studies were assessed for descriptive information and compliance with CLAIM. We grouped the individual CLAIM criteria into four domains: study description, dataset description, model description and performance description. 209 studies were included, undertaken in 37 different countries and published from 2012 to 2022. The median overall compliance of studies with all CLAIM criteria was 67% [interquartile range (IQR) 59–73%]. Median compliance was highest for the model description domain (100%, IQR 80%–100%) and substantially lower for the study description (71%, IQR 63%–86%), dataset description (63%, IQR 50%–67%) and performance description (60%, IQR 50%–70%) domains (Figure 1).

The development of an AI model requires training, in which an algorithm is exposed to data (such as CMR images) to learn features (such as where different anatomical structures are located relative to each other) that enable it to undertake a task (such as to delineate the left ventricular endocardium). This process is critical and underpins the performance and validity of all AI tools. The quality, size and variation of the dataset being used for training are of particular importance when considering the clinical applicability of a model, as a model trained on data from one population or demographic group may not generalise well when applied to others. For example, a model trained solely on CMR images from younger patients may not perform as well when used on an older population. It is essential that studies describe their data sources in a clear and transparent manner so that the generalisability of their models can be understood. This includes information about cases (such as number, eligibility criteria and clinical characteristics) and the nature of the data itself (such as the type of images and how they were acquired).

We found that although most studies indicated their data sources (94%), this was a significant omission when missing. Approximately half used publicly available datasets (49%), of which most (66%) were made available through Medical Image Computing and Computer Assisted Intervention (MICCAI) challenges, emphasising their role in advancing the field. Publicly available datasets aid reproducibility and comparison between models, but as with any retrospective data source have their own selection biases. Multiple or combined datasets were used in few studies (17%) but have the potential to improve the generalisability of models by exposure to different populations. Most studies reported the number of cases used (95%), with a median of 78 and a wide range of 3 to 12,984. Insufficient case numbers and variability are likely to affect generalisability. A minority of studies failed to report the type of CMR image used for segmentation (14%), greatly limiting the interpretability of their models.

Similarly, detailed description of the structure of AI models and the training approach are important and expected in this field. Again, this should be transparent and reproducible. Understanding the model structure can help to highlight biases in performance and thus model generalisability. However, this can be challenging due to proprietary “black-box” methodologies in design. Furthermore, publications should be written in an accessible manner such that methods are not obscured. For example, studies that present a clinical message should ensure that computer science methods and concepts (such as model structure) are explained clearly for readers who may not be AI experts (and vice versa). This balance can be difficult to achieve in such a rapidly evolving and technical field. We found that compliance with the model description domain was indeed excellent. This may reflect the fact that most were published in technical (58%) and hybrid (11%) journals. Most studies provided details about the model used (95%), training approach (78%) and software used (74%). However, open source code was only provided in a minority of studies (10%). Publishing the open source code for an AI model greatly improves transparency and facilitates the comparison of different models.

Understanding how effectively AI models perform is essential for their translation into clinical practice. Performance needs to be described in a consistent manner to enable comparison between models. However, we found that descriptions of model performance were variable, with many publications failing to present key information. The way in which performance is assessed can vary and needs to be transparent. Ideally, this should involve testing a model using a distinct and external dataset (such as images from a different population acquired by a different centre). This represents an important step in ensuring that an AI model is generalisable and valid for translation into clinical use. Only a minority (22%) of the studies that we assessed tested their models on external data. It is expected that AI models can fail, and it is good practice for studies to present an analysis of failed cases to indicate how and why this occurred. This is crucial for advancement of the field and clinical implementation. A clinician using an AI model will need to understand the factors that may predispose to false results. This goes hand-in-hand with understanding measures of diagnostic accuracy (such as sensitivity and specificity), which are major determinants of clinical utility. We noted that few studies reported failure analysis of incorrectly classified cases (32%) or estimates of diagnostic accuracy (21%).

To the best of our knowledge, this study was the largest review of the AI-based cardiac imaging literature to date. There are, of course, limitations. The review had a narrow focus on AI approaches to segmentation in CMR. Only journal papers presenting fully automated techniques were included. Semi-automated techniques incorporate both manual and AI-based elements and their distinction from fully automated techniques is open to a degree of subjectivity. The exclusion of semi-automated techniques, unpublished literature and conference abstracts were important to ensure consistent and reproducible evaluation of the included studies but did narrow the scope of the review and carried the risk of selection bias. Finally, there is an inherent risk of observer bias and interobserver variability when evaluating quality of reporting, even when using structured tools such as CLAIM; future studies may consider assessing interobserver agreement quantitatively. However, despite these limitations, our study has considered important factors for how AI studies in general are presented, and our findings are likely applicable to the broader field of AI in medical imaging.

## Discussion

**Figure 2:**
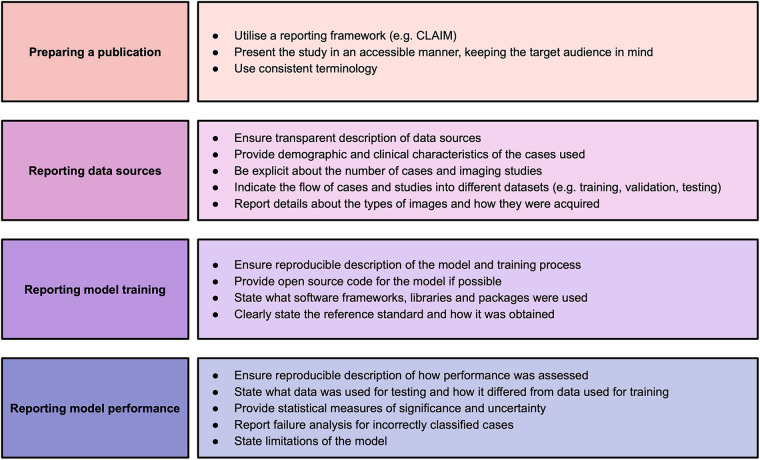
Recommendations for studies based on findings of this systematic review. Adapted from Alabed et al 2022 (13) and CLAIM (8).

This systematic review identified significant and frequent gaps in the existing literature. In this paper, we have explored some of the hallmarks of high-quality AI publications in cardiac imaging. We encourage researchers and readers to bear these in mind when presenting and appraising studies using AI methods. Based on the findings in our systematic review, we make a number of recommendations for researchers to improve the quality of reporting of AI studies, which are provided in Figure 2. Study methodology should be described in sufficient detail to enable reproducibility. Information about all data sources, including clinical characteristics of all participants, should be provided in order to understand study validity and generalisability. Testing on multiple and external datasets is an important step in the translation of AI models to clinical practice. Studies in this field may have a wide readership and publications should be accessible and transparent regardless of journal type. Tools such as CLAIM may help when presenting and reviewing studies.
